# UDP-glycosyltransferase PpUGT74F2 is involved in fruit immunity via modulating salicylic acid metabolism

**DOI:** 10.1093/hr/uhaf049

**Published:** 2025-02-18

**Authors:** Dan Jiang, Siyin Lin, Linfeng Xie, Miaojing Chen, Yanna Shi, Kunsong Chen, Xian Li, Boping Wu, Bo Zhang

**Affiliations:** Zhejiang Key Laboratory of Horticultural Crop Quality Improvement, Department of Horticulture, Zhejiang University, Zijingang Campus, 866 Yuhangtang Road, Hangzhou 310058, China; Hainan Institute of Zhejiang University, Zhenzhou Road, Sanya, Hainan 572000, China; Zhejiang Key Laboratory of Horticultural Crop Quality Improvement, Department of Horticulture, Zhejiang University, Zijingang Campus, 866 Yuhangtang Road, Hangzhou 310058, China; Melting Peach Research Institute of Fenghua District, 37 Gongyuan Road, Xikou Town, Fenghua district, Ningbo 315502, China; Zhejiang Key Laboratory of Horticultural Crop Quality Improvement, Department of Horticulture, Zhejiang University, Zijingang Campus, 866 Yuhangtang Road, Hangzhou 310058, China; Zhejiang Key Laboratory of Horticultural Crop Quality Improvement, Department of Horticulture, Zhejiang University, Zijingang Campus, 866 Yuhangtang Road, Hangzhou 310058, China; Zhejiang Key Laboratory of Horticultural Crop Quality Improvement, Department of Horticulture, Zhejiang University, Zijingang Campus, 866 Yuhangtang Road, Hangzhou 310058, China; Zhejiang Key Laboratory of Horticultural Crop Quality Improvement, Department of Horticulture, Zhejiang University, Zijingang Campus, 866 Yuhangtang Road, Hangzhou 310058, China; Collaborative Innovation Center for Efficient and Green Production of Agriculture in Mountainous Areas of Zhejiang Province, College of Horticulture Science, Zhejiang A&F University, 666 Wushu Street, Linan district, Hangzhou 311300, China; Zhejiang Key Laboratory of Horticultural Crop Quality Improvement, Department of Horticulture, Zhejiang University, Zijingang Campus, 866 Yuhangtang Road, Hangzhou 310058, China; Hainan Institute of Zhejiang University, Zhenzhou Road, Sanya, Hainan 572000, China

## Abstract

Flesh fruits are essential for human health, but pathogen infection poses a threat to fruit production and postharvest storage. The hormone salicylic acid (SA) and its metabolites, such as sugar conjugates and methyl salicylate (MeSA), play a crucial role in regulating plant immune responses. However, the UDP-glycosyltransferases (UGTs) responsible for modulating SA metabolism in fruit have yet to be identified, and further investigation is needed to elucidate its involvement in fruit immune response. Here, we identified PpUGT74F2 as an enzyme with the highest transcription level in peach fruit, responsible for catalyzing the biosynthesis of SA glucoside (SAG), but not for MeSAG formation in fruit. Furthermore, infection of peach fruit with *Monilinia fructicola*, which causes brown rot disease, led to reduced expression of *PpUGT74F2*, resulting in a significant decrease in SAG content and an increase in MeSA levels. Transgenic tomatoes expressing heterologous *PpUGT74F2* increased susceptibility to gray mold. Interestingly, overexpressing *PpUGT74F2* did not affect SA levels but dramatically reduced MeSA levels in response to pathogen infection, accompanied by significantly reduced expression of *pathogen-related* (*PR*) genes in transgenic tomatoes. This study highlights that PpUGT74F2 acts as a negative regulatory factor for fruit immunity through a distinct mechanism not previously reported in plants.

## Introduction

Flesh fruits are essential for human health due to their rich content of vitamins, minerals, dietary fiber, and antioxidants. The fruit industry has emerged as a crucial economic pillar in numerous countries and regions. However, diseases exert a profound impact on fruit yield and result in substantial economic losses both during cultivation and postharvest practices [1]. The comprehension of the underlying mechanism governing fruit disease resistance not only enriches the theoretical understanding of plant disease resistance, but also imparts valuable insights for effectively controlling fruit diseases.

In order to adapt to the environment, plants have evolved complex defense mechanisms against pathogens. Salicylic acid (SA) is a hormone and signaling molecule that plays a pivotal role in plant immune response [2–4]. SA directly triggers the accumulation of pathogenesis-related proteins (PR) and systemic acquired resistance during the pathogenesis process [5–7]. Maintaining an appropriate concentration of SA is critical because both excessive and insufficient levels can have detrimental effects on plants. Excessive accumulation of SA can exaggerate defense responses, leading to growth retardation or even cell death [8–10]. Conversely, insufficient levels of SA render plants more vulnerable to pathogens [5, 11, 12]. Upon pathogen infection, SA is synthesized through two pathways *in planta*: the PHENYLAMMONIA LYASE (PAL) pathway and the ISOCHORISMIC ACIDS SYNTHASE 1 (ICS1) pathway [13]. One important strategy to maintain a preferred concentration of SA is chemical modification, which can change the bioavailability and activity. For instance, SA can be conjugated with small organic molecules like glucose to form an inactive form that can be stored in vacuoles [14, 15].

In plant, glucose combines with hydroxyl groups to form SA glucoside (SAG), while glucose connects with carboxylate ester groups to form SA glucose ester (SGE). Both SAG and SGE were produced by UDP-glucosyltransferase (UGT). In the model organism Arabidopsis, three *UGT*s were identified to have activity to SA, including *AtUGT74F1*, *AtUGT74F2*, and *AtUGT76B1* [16–18]. Despite 77% homology and conserved active site residues, these enzymes catalyzed the formation of different products: UGT74F1 formed SAG, while UGT74F2 mainly formed SGE [16]. Lower levels of SA and resistance to bacterial infection from *Pseudomonas syringae* were found in *ugt74f1* mutants. However, *ugt74f2* mutants had higher levels of SA and increased resistance to *P. syringae*, whereas overexpression of *UGT74F2* leads to lower levels of SA and enhanced susceptibility to *P. syringae* [18, 19]. The mutant *ugt74e2* also had higher resistance to *P. syringae*, which showed enhanced systemic immunity during SAR, and increased levels of *pathogen-related 1* (*PR1*) and *PR5* transcripts [20]. AtUGT76B1 is responsible for production of SAG, and *ugt76b1* mutant exhibited enhanced resistance to *P. syringae* pv. *tomato* (*Pst*) DC3000 *avrRpm1* with an increased SA content compared with wild type (WT) [17]. Recently, the SA carboxyl glucosyltransferase CsUGT87E7 from *Camellia sinensis* has been found to regulate SA homeostasis. After infection, the accumulation of SGE and SA in *CsUGT87E7*-silenced plants significantly reduced, resulting in the expression of *PR* genes declining and the resistance of tea plants decreasing [21]. These studies revealed that the homeostasis of SA could be affected by UGT, and then correspondingly regulated the resistance of plant.

SA regulates plant immune responses in concert with other molecules, such as methyl salicylate (MeSA), which is a common ester derivative of SA synthesized by the enzyme SA methyltransferase SAMT1 (SA methyl transferase I) [22, 23]. MeSA functions as a mobile signal during systemic acquired resistance, inducing defense against pathogens, and also serves as an airborne signal for plant-to-plant communication and defense against aphids and viruses [24–27]. Tomato fruits with high content of MeSA had enhanced defense response to postharvest diseases [28]. The enzyme AtUGT71C3 catalyzes the conversion of MeSA to MeSA glucoside (MeSAG) [29]. Compared to the WT, the *ugt71c3* mutant elevated levels of systemic MeSA and SA, resulting in enhanced resistance to *Pst* DC3000. The glucosylation mediated by UGT71C3 thus decreases the level of MeSA and facilitates negative regulation of plant systemic acquired resistance, emphasizing the importance of maintaining homeostasis of MeSA and SA in response to biotic stresses.

The combined production of top 10 fruit crops, including tomato (*Solanum lycopersicum*) and peach (*Prunus persica L*. Batsch), which amounts to >630 million tons, accounts for ~46% of global fruit production (FAOSTAT, http://www.fao.org/faostat/). Peaches have been found to be highly susceptible to fungal pathogens such as *Monilinia spp*., especially *Monilinia fructicola*, causing brown rot disease in fruits [30]. Brown rot disease seriously deteriorates fruit quality, especially affecting storage and preservation of peach fruit [31]. Tomato gray mold caused by *Botrytis cinerea* pathogen and bacterial leaf spot caused by *Pst* DC3000 are both the most serious diseases on tomatoes, causing serious losses to fruit yield and quality [32]. Up to now, research on the plant disease resistance mechanism has predominantly focused on the physiological functions of SA in its free form as a hormone, while the role of glycosylation metabolism mediated by UGTs in disease resistance remains unexplored in fruits.

Here, an SA glycosyltransferase PpUGT74F2 was cloned and functionally identified. Enzymatic assays in *vitro* and altered expression in fruit indicated that PpUGT74F2 could catalyze the formation of SAG, not MeSAG. Furthermore, *PpUGT74F2* was inhibited after infection with *M*. *fructicola* in peach, to promote the accumulation of SA and MeSA. Notably, the transgenic tomato expressing *PpUGT74F2* exhibited reduced immunity against *Pst* DC3000 and *B. cinerea*. Our study has enhanced the comprehension of the transformation between SA and SAG following fungal infection in fruits, while also shedding light on the role of SA glycosyltransferase during plant pathogen infection.

## Results

### The *in vitro* analysis of enzyme activity for the recombinant protein PpUGT74F2

**Figure 1:**
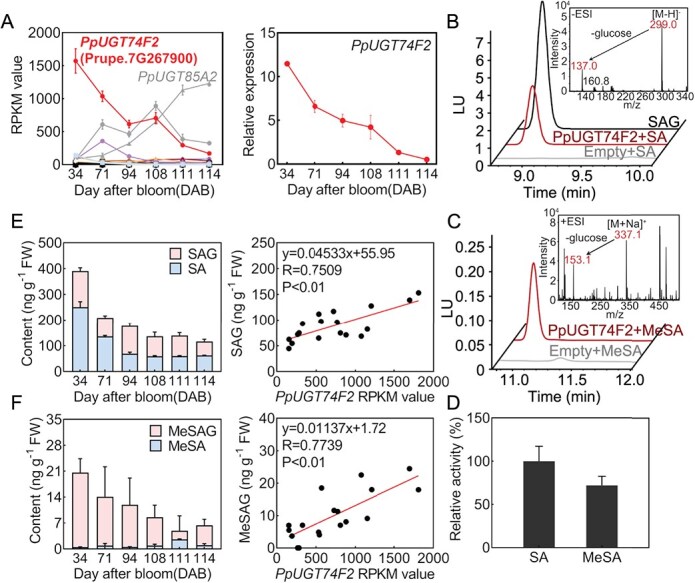
The relationship of *PpUGT74F2* with SA and MeSA. **A**, Expression levels of *PpUGT*s in six developmental stages of peach fruit by RNA-sequencing and RT-qPCR analysis for *PpUGT74F2* expression during peach fruit development. **B**, Enzymatic activity analysis of PpUGT74F2 toward SA. **C**, Enzymatic activity analysis of PpUGT74F2 toward MeSA. **D**, Relative enzyme activities of PpUGT74F2 with SA and MeSA. **E**, The content of SA and SAG in six developmental stages of peach fruit and correlation analysis between SAG and *PpUGT74F2*. **F**, The content of MeSA and MeSAG in six developmental stages of peach fruit and correlation analysis between MeSAG and *PpUGT74F2*. For **A**, **D**, **E**, and **F**, data are presented as mean ± SD (*n* = 3).

RNA-sequencing results revealed expression profiles of 163 members in UGT gene family during peach fruit development and ripening [33]. Among these *UGT* genes, Prupe.7G267900 (*PpUGT74F2*) exhibited a declining expression pattern, and had the highest transcript levels when compared to other UGT genes at young fruit stage (Fig. 1A, Supplementary Data Table S1). Real time quantitatvie PCR (RT-qPCR) results confirm the gene expression pattern for *PpUGT74F2* (Fig. 1A). In contrast, *PpUGT85A2* showed the highest expression level at fruit ripe stage, exhibiting an increased expression pattern during fruit development and ripening. Our previous study characterized that PpUGT85A2 catalyzes the glucosylation of linalool in peach fruit [34]. To investigate the role of Prupe.7G267900 in peach fruit, phylogenetic analysis was performed. PpUGT74F2 belongs to group L in the plant UGT family [33], and was clustered together with Arabidopsis AtUGT74F1 and AtUGT74F2 (Supplementary Data Fig. S1), which are responsible for catalyzing SA glycosylation. Amino acid sequence analysis revealed that PpUGT74F2 shared 51.5% identity with AtUGT74F1 and 53.6% with AtUGT74F2, respectively (Supplementary Data Table S2).

To investigate the physiological role of PpUGT74F2, it was cloned and expressed in *Escherichia coli*. The UDP-glucose was used as sugar donor, and 29 chemicals were tested as potential substrates of PpUGT74F2, including 7 plant hormones (SA, IAA, JA, ABA, BR, ZT, IBA), 13 flavonoids (quercetin, myricetin, kaempferol, isorhamnetin, catechins, epicatechin, naringin, hesperidin, luteolin, celery, baicalein, genistein, daidzein), 7 volatiles (α-Terpineol, linalool, geraniol, eugenol, 2-phenylethanol, benzyl alcohol, MeSA), and 2 anthocyanins (mallow pigment, cornflower) (Supplementary Data Table S3). The *in vitro* enzymatic activity analysis revealed that PpUGT74F2 demonstrated activity toward SA and MeSA, leading to the formation of SAG and MeSAG, respectively (Fig. 1B and C). Liquid chromatography-mass spectrometry (LC–MS) confirmed the enzymatic reaction products, with product peaks at m/z 299 [M−H]^−^ identified as SAG and 337 [M + Na]^+^ was identified as MeSAG. Notably, the activity of PpUGT74F2 toward MeSA was found to be 28% lower compared to its activity toward SA (Fig. 1D).

### The analysis of relationship between *PpUGT74F2* and SA metabolites

In peach fruit, SA glucose is present predominantly as SAG, accounting for 92.15%, but not SGE (Supplementary Data Fig. S2). To elucidate the relationship between *PpUGT74F2* and SAG, the content of SAG was detected during peach fruit ripening. Approximately 35.84% of total SA in young peach fruit harvested at 34 days after bloom (DAB) was found as SAG (Fig. 1E). As the fruit developed and matured, the level of SAG gradually reduced along with a decline in *PpUGT74F2* expression. Linear regression analysis revealed a strong positive correlation between *PpUGT74F2* expression and the accumulation of SAG (R = 0.75, *P* < 0.01). Furthermore, there was a significant positive correlation between the content of SAG and SA (R = 0.68, *P* < 0.01) (Supplementary Data Fig. S3). Meanwhile, we measured the levels of MeSA during peach fruit ripening (Fig. 1F). The content of MeSAG was significantly higher compared to that of MeSA in peach fruit and positively correlated with the expression of *PpUGT74F2* (R = 0.77, *P* < 0.01) (Fig. 1F).

**Figure 2:**
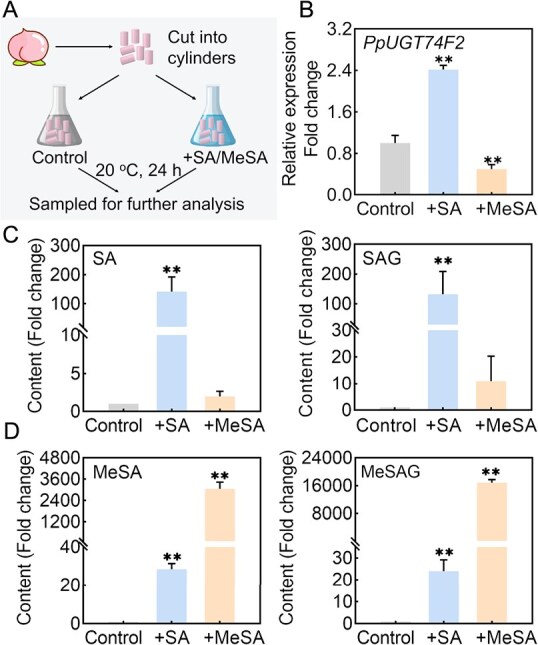
Changes in *PpUGT74F2* expression and levels of SA, MeSA, SAG, and MeSAG in peach fruit following SA and MeSA treatments. **A**, Schematic representation of the experimental design for exogenous SA and MeSA treatments in peach fruit. **B**, Quantitative analysis of *PpUGT74F2* expression in peach fruit. **C**, Content of SA and SAG in peach fruit. **D**, Measurement of MeSA and MeSAG levels in peach fruit. Data are presented as mean ± SD (*n* = 3). Significant differences were presented with asterisks (Student’s *t*-test, ^**^P < 0.01).

To further determine the relationship between *PpUGT74F2* and SA metabolites, peach fruit was treated with exogenous SA and MeSA (Fig. 2A). A significant increase in the content of SA was observed in peach fruit treated with SA. It was found that *PpUGT74F2* expression was induced by SA, leading to substantial accumulation of SAG (Fig. 2B and C). In addition, there was a robust increase in the levels of MeSA and MeSAG after SA treatment (Fig. 2D). Furthermore, the expression of *PpUGT74F2* was inhibited by MeSA (Fig. 2B). Compared to the control, treatment with MeSA resulted in elevated levels of both SA and SAG, accompanied by a significant increase in the content of both MeSA and MeSAG (Fig. 2C and D).

### PpUGT74F2 catalyzes the formation of SAG but not MeSAG *in vivo*

**Figure 3:**
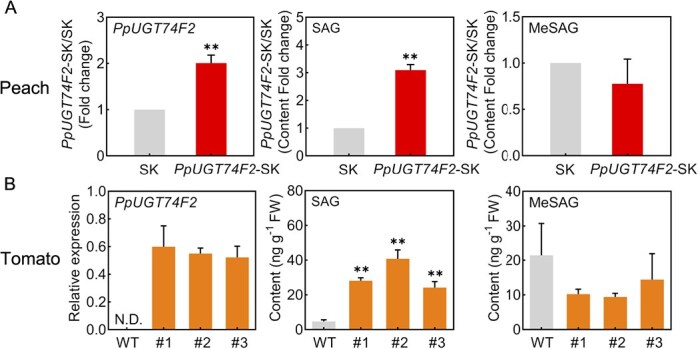
Expression of *PpUGT74F2* modulate the level of SAG in fruit. **A**, Expression of *PpUGT74F2*, content of SAG and MeSAG in peach fruit after overexpressing *PpUGT74F2*. Data are from three biological replicates. Significant differences were presented with asterisks (Student’s *t*-test, ^**^P < 0.01). **B**, Expression of *PpUGT74F2* and content of SAG and MeSAG in transgenic tomato fruit. Data are presented as mean ± SD (*n* = 3). N.D., not detected. Significant differences were presented with asterisks (Student’s *t*-test, ^**^P < 0.01).

Currently, achieving transgenic technology in peach fruits remains challenging. To investigate the *in vivo* biological function of PpUGT74F2, homologous transient overexpression experiments were conducted. Overexpression of *PpUGT74F2* in peach fruits promoted significantly enhanced SAG accumulation (~3-fold change), while MeSAG showed no significant alternation (Fig. 3A).

Additionally, heterologous stable overexpression *PpUGT74F2* under the control of 35S promoter was performed in tomato fruit (Fig. 3B). The transgenic tomatoes exhibited no differences in pigmentation, ethylene production and fruit firmness compared to the WT (Supplementary Data Fig. S4), indicating that heterologous expression of *PpUGT74F2* does not affect the ripening process of tomato fruit. Notably, transgenic tomato fruit produced ~6-, 9-, and 5-fold higher levels of SAG content than those in WT across three different lines. Conversely, no significant changes were observed in MeSAG content in transgenic tomato fruit (Fig. 3B). These findings confirm that *PpUGT74F2* is responsible for the synthesis of SAG rather than MeSAG both in peach and tomato fruit.

### Infection of *M. fructicola* suppresses *PpUGT74F2* expression and modulates SA metabolism in peach fruit

**Figure 4:**
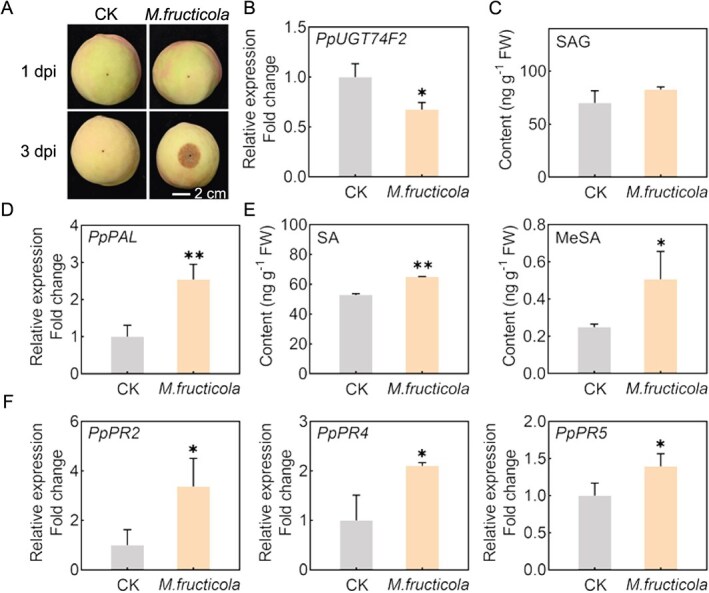
Effects of *M. fructicola* infection on expression of *PpUGT74F2* and content of SA and MeSA. **A**, Photographs of peach infected by *M. fructicola*. The scale bar is consistent across each image. Expression of (**B**) *PpUGT74F2*, (**D**) *PpPAL*, and (**F**) *PpPR*s in peach fruit infected by *M. fructicola* after 3 dpi. Content of (**C**) SAG, (**E**) SA and MeSA in peach fruit infected by *M. fructicola* after 3 dpi. For **B**–**F**, data are presented as mean ± SD (*n* = 3). Significant differences are indicated with asterisks (Student’s *t*-test, ^*^P < 0.05, ^**^P < 0.01).

The involvement of SA in plant stress defense against pathogens is well established, but the specific role of UGT in SA glycosylation and its impact on defense responses in fruit crops remains unknown. Among the various diseases affecting peach fruit, brown rot caused by the *M. fructicola* pathogens leads to the greatest losses. Therefore, we conducted an experiment where peach fruit was inoculated with *M. fructicola* pathogens to investigate the relationship between *PpUGT74F2* and immune response (Fig. 4A). Our findings revealed that expression of *PpUGT74F2* is repressed upon *M. fructicola* infection in peach fruit after 3 days postinoculation (dpi) (Fig. 4B). Interestingly, no significant changes in SAG content were observed after infection with *M. fructicola* (Fig. 4C). Notably, expression of *PpPAL*, responsible for SA synthesis, was induced by *M. fructicola*, causing a significant accumulation in the content of SA (Fig. 4D and E). The content of SAG and SA exhibited a positive correlation (*R* = 0.68, *P* < 0.01) in peach fruit (Supplementary Data Fig. S3). Consequently, the levels of SAG did not decrease following infection with *M. fructicola* due to the accumulation of SA. Furthermore, infection with *M. fructicola* led to a slight upregulation in expression of *PpSAMT* following (Supplementary Data Fig. S5). The combined increase in both SA levels and *PpSAMT* expression resulted in an approximate 2-fold elevation in MeSA content (Fig. 4E). PRs are defense proteins that positively regulate the inhibition of pathogen development and transmission in plants [35]. The present results demonstrated a significant upregulation of *PpPR2*, *PpPR4*, and *PpPR5* expression following inoculation (Fig. 4F).

**Figure 5:**
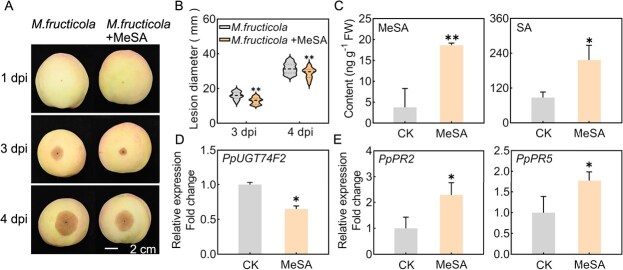
MeSA suppressed *PpUGT74F2* expression to inhibit the disease in peach fruit. **A**, Photograph of peach after infection by *M. fructicola* with application of MeSA. The scale bar is consistent across each image. **B**, Lesion diameter of peach fruit treated with MeSA and inoculated with *M. fructicola*. The dashed lines denote median and the dotted lines represent quartiles. Data were collected from 20 to 30 peach fruits. Significant differences are indicated with asterisks (Student’s *t*-test, ^**^P < 0.01). **C**, The content of MeSA and SA in peach fruit treated with MeSA and inoculated with *M. fructicola* (4 dpi). **D**, The expression of *PpUGT74F2* and (**E**) *PpPR2* and *PpPR5* in peach fruit treated with MeSA and inoculated with *M. fructicola* (4 dpi). For **C**, **D,** and **E**, data are presented as mean ± SD (*n* = 3). Significant differences are indicated with asterisks (Student’s *t*-test, ^*^P < 0.05, ^**^P < 0.01).

In addition, exogenous treatment of MeSA was beneficial for inhibiting the growth of *M. fructicola* in peach fruit (Fig. 5A and B), owing to a significant increase in the levels of MeSA and SA following MeSA application (Fig. 5C). Notably, the expression of *PpUGT74F2* was suppressed after MeSA treatment in peach fruit infected with *M. fructicola* (Fig. 5D). The observed increase in *PpUGT74F2* expression following MeSA treatment is consistent with the findings presented in Fig. 2. Meanwhile, MeSA induced the expression of disease resistance genes (*PpPR2* and *PpPR5*) (Fig. 5E). These results suggest that suppression of *PpUGT74F2* expression facilitates enhanced accumulation of SA and MeSA, thereby reinforcing the resistance of peach fruit against pathogenic invasion.

### Heterologous expression of *PpUGT74F2* enhanced susceptibility to *Pst* DC3000 in tomato leaf

**Figure 6:**
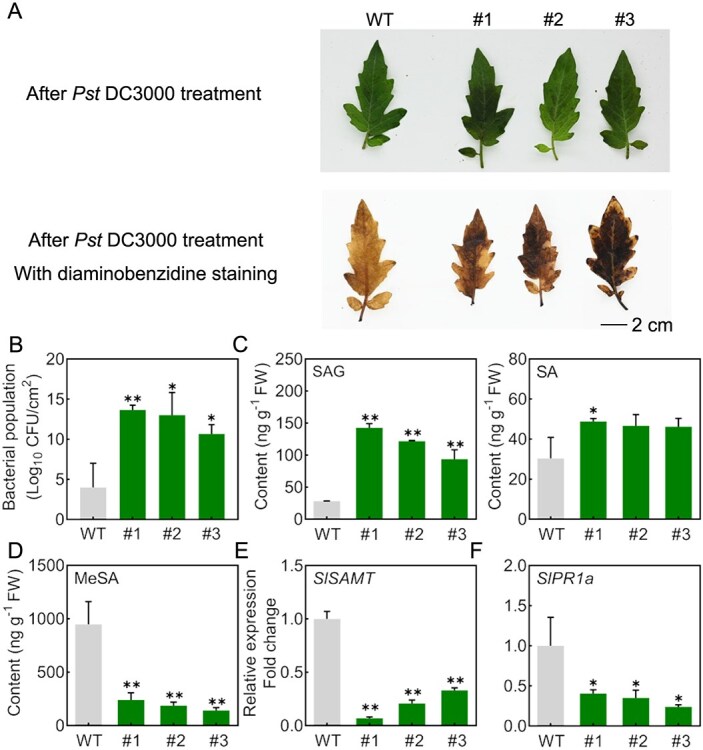
The heterologous expression of *PpUGT74F2* in tomato enhances susceptibility to *Pst* DC3000. **A**, Photographs of transgenic tomato leaves after infection with *Pst* DC3000, with and without diaminobenzidine staining (2 dpi). The scale bar is consistent across each image. **B**, Growth of *Pst* DC3000 in the leaves infected with pathogens. Data are from three biological replicates with three leaves each and presented as mean ± SD. Significant differences are indicated with asterisks (Student’s *t*-test, ^*^P < 0.05, ^**^P < 0.01). **C**, The content of SAG and SA in transgenic tomato leaves with *Pst* DC3000 infection after 2 dpi. **D**, The content of MeSA in transgenic tomato leaves with *Pst* DC3000 infection after 2 dpi. **E**, The expression of *SlSAMT* and (**F**) *SlPR1a* in transgenic tomato leaves with *Pst* DC3000 infection after 2 dpi. For **B**–**F**, data are presented as mean ± SD (*n* = 3). Significant differences are indicated with asterisks (Student’s *t*-test, ^*^P < 0.05, ^**^P < 0.01).

To further elucidate the role of *PpUGT74F2* in plant immunity, we inoculated transgenic tomato leaves with *Pst* DC3000. After infection by *Pst* DC3000, diaminobenzidine staining was performed to detected H_2_O_2_ levels. Transgenic tomato leaves exhibited more severe symptoms than WT (Fig. 6A). Furthermore, the population of bacterial communities on transgenic tomato leaves was significantly higher than that on WT leaves by >3-fold (Fig. 6B).

To investigate whether the susceptibility of the transgenic tomatoes to pathogens is attributed by an alternation in levels of SA and its metabolites, we quantified the content of SA, SAG, and MeSA. The accumulation of SAG is attributed to the expression of *PpUGT74F2* (Fig. 6C). The levels of SA accumulated in transgenic tomato leaves infected with *Pst* DC3000 were comparable to that observed in WT plants (Fig. 6C). It is noteworthy that there was a significant decrease in MeSA levels in transgenic tomato leaves (Fig. 6D). Expression levels of *SlSAMT*, involved in converting SA to MeSA, were significantly reduced following pathogen infection (Fig. 6E). Meanwhile, *SlPR1a* and *SlPR1b* expression was significantly downregulated upon infection with *Pst* DC3000 (Fig. 6F and Supplementary Data Fig. S6). These results indicate that heterologous expression of *PpUGT74F2* enhanced susceptibility to *Pst* DC3000.

### Heterologous expression of *PpUGT74F2* enhanced susceptibility to *B. cinerea* in tomato fruit

**Figure 7:**
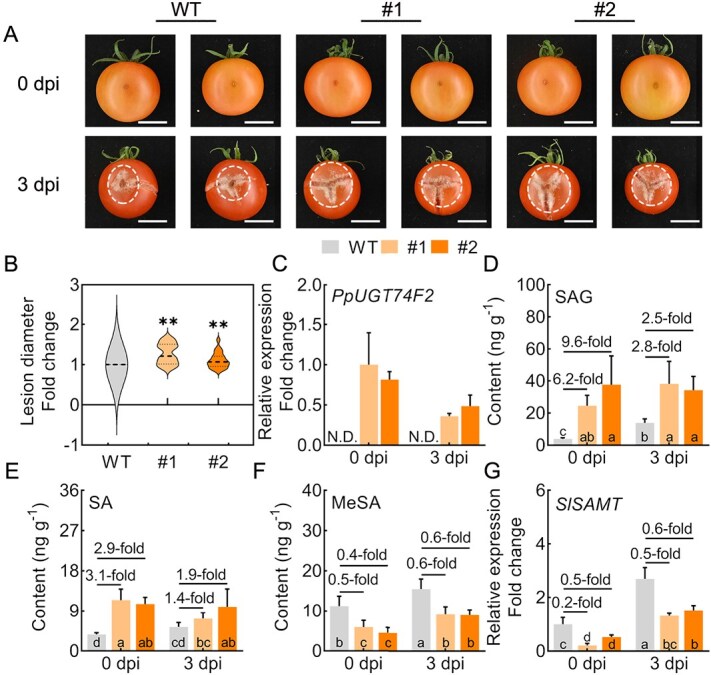
Transgenic tomato fruit enhances susceptibility to *B. cinerea*. **A**, Photos of transgenic tomato fruit following *B. cinerea* infection at 0 and 3 dpi. Scale bar = 2 cm. **B**, Lesion diameter of tomato fruit after *B. cinerea* infection at 3 dpi. Data were collected from 21 to 27 tomato fruits for each transgenic line and WT plants. The dashed lines denote median and the dotted lines represent quartiles. Significant differences are indicated with asterisks (Student’s *t*-test, ^**^P < 0.01). The expression of (**C**) *PpUGT74F2* and (**G**) *SlSAMT* in transgenic tomato fruit with *B. cinerea* infection at 0 and 3 dpi. The content of (**D**) SAG, (**E**) SA, and (**F**) MeSA in transgenic tomato fruit with *B. cinerea* infection at 0 and 3 dpi. For **C**–**G**, Data are presented as mean ± SD (*n* = 3). Fold changes are presented above the bars. Significant differences are indicated with different letters in the bars at *P* < 0.05 (Tukey one-way ANOVA).

The transgenic tomatoes exhibited increased susceptibility to *B. cinerea* compared to WT fruit, as evidenced by significantly larger lesion diameters on transgenic tomato fruit (Fig. 7A and B). It is worth noting that the expression levels of *PpUGT74F2* tended to decrease after 3 days of infection (3 dpi) in the transgenic tomato (Fig. 7C). The SAG content in the transgenic tomato fruit increased compared to the WT. However, the magnitude of increase decreased with prolonged pathogen infection (Fig. 7D). A similar trend was observed for SA content in the transgenic tomato following a 3-day infection with *B. cinerea* (Fig. 7E).

Consistent with the decreased content of MeSA (Fig. 7F), a significant reduction in transcript levels was observed for *SlSAMT* in transgenic tomato fruit infected by *B. cinerea* at both 0 and 3 dpi (Fig. 7G). Notably, the fold changes in SAG, SA, and MeSA content at 3 dpi with *B. cinerea* infection were lower than those at 0 dpi. For instance, at 3 dpi with *B. cinerea* infection, the content changes of SAG in transgenic tomato line #1 were 2.8-fold, whereas at 0 dpi, the content changes of SAG were 6.2-fold. It is likely that this could be attributed to the suppression of *PpUGT74F2* expression in transgenic tomatoes following infection by *B. cinerea* at 3 dpi compared to the expression at 0 dpi (Fig. 7C). Meanwhile, there was a significant decrease in *SlPR1a* and *SlPR1b* expression after infection by *B. cinerea* at 3 dpi (Supplementary Fig. S7). These results demonstrate that *PpUGT74F2* negatively regulates fruit disease resistance.

## Discussion

### PpUGT74F2 is an enzyme associated with SA glucosylation in fruit

As a crucial hormone, SA exists in both free and conjugated forms in plants, playing pivotal roles in growth and disease resistance [36]. Although UGTs have been reported to catalyze the glycosylation of SA in plants, no UGTs have been identified to be associated with SA metabolism in fruit crops. In this study, we discovered that SAG is the predominant SA glycosylation product in peach fruit, accounting for ~92.15% of total. Enzymatic activity analysis toward 29 chemicals, including 8 hormones, 7 volatiles, 13 flavonoids, and 2 anthocyanins, demonstrated that recombinant protein PpUGT74F2 catalyzes the glucosylation of SA and MeSA, using UDP-glucose as a sugar donor to produce SAG and MeSAG *in vitro*, respectively (Fig. 1D). However, the expression of *PpUGT74F2* was induced by SA treatment in peach fruit, whereas its expression was inhibited by MeSA. Furthermore, overexpression of *PpUGT74F2* in peach and tomato fruits led to a significant increase in SAG levels, but did not elevate the levels of MeSAG *in vivo* (Fig. 3). Our findings indicate that PpUGT74F2 catalyzes the glycosylation of SA but not MeSA in fruit. To the best of our knowledge, no UGT member has been reported to catalyze the glucosylation of both SA and MeSA in plants.

The phylogenetic analysis indicated that peach PpUGT74F2 clustered closely with Arabidopsis AtUGT74F1 and AtUGT74F2, displaying amino acid identities of 51.5% and 53.6%, respectively (Supplementary Data Fig. S1 and Table S2). However, PpUGT74F2 exhibits only 22.1% sequence identify with AtUGT76B1, despite the latter enzyme being responsible for SAG formation (Supplementary Data Table S2). These results suggest the involvement of multiple UGT enzymes in regulating SA metabolism. Furthermore, it is crucial to recognize that sequence homology does not necessarily indicate functional similarity among UGTs. Therefore, both *in vitro* and *in vivo* experiments are necessary to clarify UGT roles in fruit crops.

Plant hormones are essential for fruit development and stress responses. Previous studies have demonstrated that SlUGT75C1 catalyzed the glucosylation of ABA, and the suppression of *SlUGT75C1* expression significantly accelerates fruit ripening in tomatoes by increasing ABA levels and enhancing ethylene production [37]. Beyond ABA, the present study indicates that SA can also be glucosylated by PpUGT74F2, thereby broadening our knowledge of plant hormone metabolism in fruit crops.

### The negative regulatory role of PpUGT74F2 in disease resistance

In the present study, the infection of *M. fructicola* led to a decrease in the expression levels of *PpUGT74F2* in peach fruit, concomitant with a significant increase in SA content and the expression of *PR* genes (Fig. 4). Notably, transgenic tomatoes with heterologous expression of *PpUGT74F2* displayed heightened susceptibility to *Pst* DC3000 in leaves and *B. cinerea* in fruits (Fig. 6A and Fig. 7A). These findings suggest that *PpUGT74F2* functions as a negative regulator of plant disease resistance, as evidenced in both peach and tomato. Previous studies have demonstrated that UGTs modulate disease resistance through their impact on SA levels in plants. For instance, Arabidopsis *ugt74f2* mutant displays the enhanced resistance against *P. syringae* due to elevated SA levels [16, 18,]. Similarly, AtUGT76B1 is capable of producing SAG, and the *ugt76b1* mutant exhibits increased SA content and enhanced disease resistance to *Pst* DC3000 in Arabidopsis [17]. Consequently, *AtUGT74F2* and *AtUGT76B1* are recognized as negative regulators of immune response in Arabidopsis, consistent with the findings for peach *PpUGT74F2* presented herein.

Contrastive immune responses have been observed for UGTs involved in SA glycosylation. For instance, Arabidopsis AtUGT74F1 catalyzes the formation of SAG, yet the *ugt74f1* mutant exhibited increased susceptibility to *P. syringae* due to reduced SA levels [18]. Moreover, silencing *CsUGT87E7* in tea leaves significantly reduces the accumulation of SA after infection, thereby enhancing susceptibility to pathogen infection [21]. Furthermore, AtUGT76D1 catalyzed the glycosylation of 2,3-dihydroxybenzoic acid (2,3-DHBA) and 2,5-DHBA, which are primary catabolic products of SA. Overexpression of *AtUGT76D1* leads to elevated SA levels and significant upregulation of *PR* genes [38]. Collectively, these findings indicate that plant UGTs exert both positive and negative regulatory influences on plant immune response, contingent upon their impact on SA levels. Notably, the present study did not observe a significant alternation in SA content in transgenic tomatoes expressing heterologous *PpUGT74F2*, despite an observed increase in susceptibility to pathogen infection (Fig. 6 and Fig. 7). Therefore, we conducted experiments to elucidate the mechanism by which PpUGT74F2 modulates immune response.

In addition to SAG and SGE, SA can also undergo methylation catalyzed by SAMT enzyme to form MeSA, which functions as a mobile signaling molecule in plant immune response [24, 39–43]. In our present study, the application of exogenous MeSA to peach fruit led to an increase in the expression of *PR* genes and effectively inhibited *M. fructicola* occurrence (Fig. 5). Following pathogen infection, a significant decline in MeSA levels and reduced resistance were observed in transgenic tomato leaves and fruits (Fig. 6 and Fig. 7). Notably, the SA content in transgenic fruit gradually returned to levels comparable to those of WT fruit upon *B. cinerea* invasion, while the MeSA content in transgenic tomatoes remained consistently lower than that of WT. Tomato fruit with elevated MeSA levels exhibited reduced susceptibility to common postharvest infections such as *B. cinerea* [28]. The decreased expression of *SlSAMT1* is associated with the decline in MeSA levels subsequent to pathogen infection (Fig. 6E, Fig. 7G). These findings suggest that the reduction in MeSA levels, resulting from decreased *SlSAMT* expression in transgenic tomatoes expressing *PpUGT74F2*, is a major factor influencing the immune response.

Our present observations indicate that PpUGT74F2 specifically catalyzes the glycosylation of SA, but not MeSA glycosylation *in planta*. Additionally, pathogen infection results in a reduction of *PpUGT74F2* transcript levels, coinciding with an elevation in MeSA levels in both peach and tomato fruits. A negative correlation between MeSA content and *PpUGT74F2* transcript levels was also observed in peach fruit (Supplementary Fig. S8). To determine whether this mechanism is conserved across plant species, it would be intriguing to evaluate MeSA levels in Arabidopsis *UGT* mutants and transgenic plants following pathogen infection. Furthermore, investigating the mechanism by which increased *PpUGT74F2* expression influences *SAMT* transcript levels in fruit would provide deeper insights into this process.

In conclusion, by integrating transcriptome data, *in vitro* enzyme activity assays, and overexpression studies in fruit, we have identified that PpUGT74F2 is responsible for catalyzing the glucosylation of SA. The expression of *PpUGT74F2* was observed to decrease in response to various pathogens, such as *M. fructicola* in peach fruit and *B. cinerea* in tomato fruit. Heterologous expression of *PpUGT74F2* in transgenic tomatoes resulted in increased susceptibility to pathogen infection. Our results demonstrate that PpUGT74F2 functions as a negative regulator of plant disease resistance through an unprecedented mechanism, thus expanding the known roles of UGTs in horticultural crops.

## Materials and methods

### Plant materials

Fruit of peach cultivars ‘Hujingmilu’ were harvested from the Melting Peach Research Institute, Ningbo City, Zhejiang Province, China. Peach fruit undergoes six developmental stages prior to sampling according to previous study [33]. Each sample consists of five fruits with three biological replicates, and fruit samples that have been completely frozen with liquid nitrogen are stored at −80°C.

### Measurements of SA and its glucosylation products

SA was extracted using the method with modification as previously described [44]. The glycoside SAG was extracted by SPE LC-18 column (CNW, Duesseldorf, Germany) and hydrolyzed by AR2000 (Rapidase, Séclin, France) according to a previous study [33]. The mixture was dissolved in 5% (v/v) trichloroacetic acid after being blow dried by nitrogen gas. Then released SA from SAG was extracted by ethylacetate-cyclopentane, blow dried by nitrogen gas, dissolved in methanol, and analyzed by high performance liquid chromatography (HPLC). The conditions for HPLC were the same as previously described [44].

### Purification of recombinant protein and analysis of enzymatic activity

The pGEX-GST-*PpUGT74F2* vector was constructed and the protein was purified using the method as previously described [44]. For analysis of enzymatic activity, reactions were carried out in 50 μl of reaction mixture, consisting of 50 mM Tris–HCl buffer (pH 6.0), 1 mM UDP-glucose, 1 mM substrates, and 2.5 μg recombinant protein. The mixture was reacted at 30°C for 30 min, and terminated by methanol. Enzyme products were analyzed by HPLC and confirmed by LC–MS. The conditions for HPLC and LC–MS were the same as the previous study [33].

### Gene expression analysis

RNA was isolated from peach and tomato fruit following the method described in the previous study [45]. The cDNA was synthesized by the kit of Hiscript II Q RT SuperMix (Vazyme, China). qPCR analysis was carried out via ChamQ Universal SYBR qPCR Master Mix (Vazyme, China) and performed on CFX96 instrument (Bio-Rad). Primers for qPCR are described in Supplementary Data Table S5. The preparation of libraries for high-throughput Illumina strand-specific RNA-Seq was carried out as previously reported [46]. Analysis of gene expression was conducted with three biological replicates.

### Transient overexpression in peach fruit

The pGreen-*PpUGT74F2*-SK vector was constructed with the primers listed in Supplementary Data Table S4, and transformed into *Agrobacterium tumefaciens* strain GV3101:: pSoup. Transient overexpression in peach fruit was performed according to the previous study [47]. The vector containing *PpUGT74F2* and empty vector were separately introduced into *A. tumefaciens*, which were then introduced into opposite sides of 1 cm thick flesh cubes obtained from the same fruit. After infection, flesh cubes were washed with sterile water, transferred to MS medium, and incubated for 3 days at 20°C. Flesh cubes were sampled for further analysis. The experiment was conducted three times.

### Transgenic tomato obtained and growth

The pBI121-*PpUGT74F2* vector with CAMV35S promoter was constructed. Transformation of Ailsa Craig tomato was carried out following the previous study [48]. Tomato plants were grown in a greenhouse (25°C, 16/8 h, light/dark). Tomato fruit were harvested at 7 days after breaker (B + 7). Each line includes three biological replicates.

### The infection of pathogen assays in peach and tomato

For fruit inoculation, the experiment was conducted following the procedures of the previous study [44]. For tomato plant inoculation with *Pst* DC3000, the method was referred to the previous study [49]. Briefly, *Pst* DC3000 was grown on the King’B medium (pH 7.2), which consist of 20 g peptone, 20 ml glycerol (V/V, 50%), 1.5 g K_2_HPO_4_, 15 g agar, 6 ml MgSO_4_ (1 M), 2 ml rifampicin (10 mg ml^−1^), and 1 l H_2_O. Tomato plants were infected with solution containing *Pst* DC3000 (OD_600_ = 0.0002) and MgCl_2_ (10 mM), then grown at 24°C under high humidity conditions. Leaves were collected for phenotypic observation and further study after 2 dpi. The experiments were designed with three biological replicates.

### Phylogenetic analysis

The amino acid sequences were obtained from https://www.ncbi.nlm.nih.gov/ and https://phytozome-next.jgi.doe.gov/. The phylogenetic tree was generated by MEGA software (version 7.0, USA), utilizing the neighbor-joining (NJ) method with 1000 bootstrap. Va5GT, AHL68667; FaGT2, AY663785; MdUGT75B1, XP_008380456; CsUGT75L12, ALO19892; UGT84A13, AHA54051; CsUGT84A22, ALO19890; AtUGT75B1, AT1G05560; AtUGT75B2, AT1G05530; AtUGT74F1, AT2G43840; AtUGT74F2, AT2G43820; AtUGT74E2, AT1G05680; AtUGT84A2, AT3G21560; AtUGT84B1, AT2G23260.

### Statistical analysis

Figures were prepared via GraphPad Software (San Diego, CA). Multiple groups significance test was calculated using Tukey one-way analysis of variance (ANOVA), and the two-sample significance test was conducted using Student’s *t-*test (SPSS Inc., Chicago, IL, USA). *P* < 0.05 was considered to indicate statistical significance.

## Supplementary Material

Web_Material_uhaf049

## Data Availability

RNA-Seq raw data can be found in the NCBI with accession number PRJNA576753 for peach samples at different development and ripening stages.
