# Supplementary material for: UDP-glycosyltransferase PpUGT74F2 is involved in fruit immunity via modulating salicylic acid metabolism
Source: Hortic Res. 2025 Feb 18;12(6):uhaf049. doi: 10.1093/hr/uhaf049 (PMC12010879; doi:10.1093/hr/uhaf049)
Supplement: Web_Material_uhaf049 [file web_material_uhaf049.zip › Supplementary files.docx]

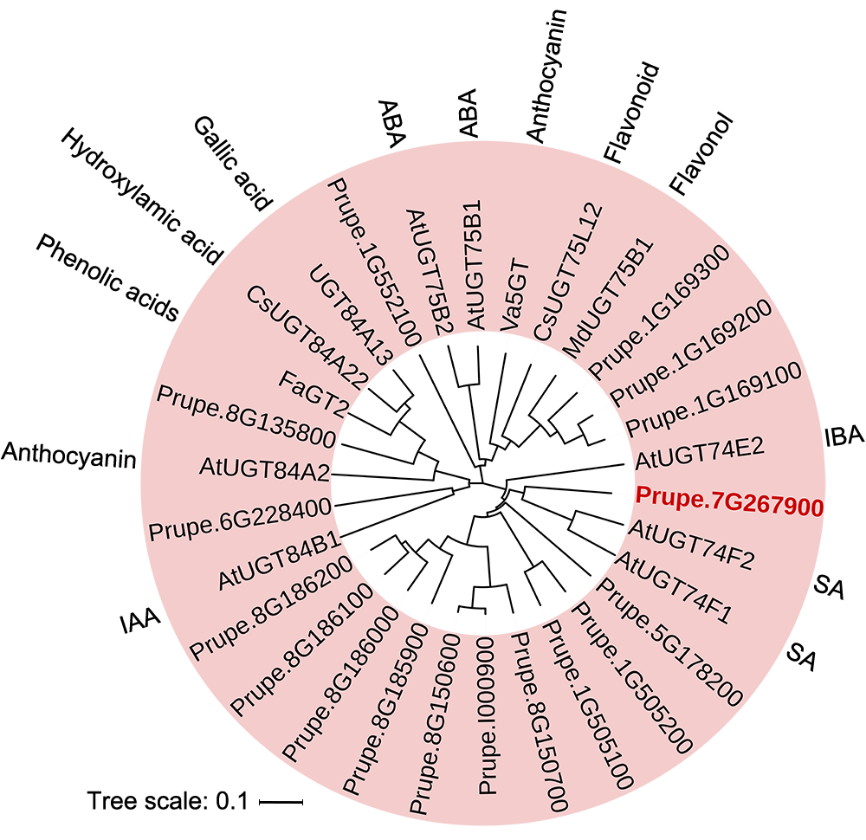


**Figure S1.** A phylogenetic tree for UGTs from group L.


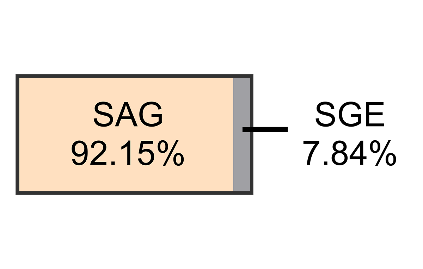


**Figure S2.** The relative proportions of SAG and SGE in peach fruit.

**
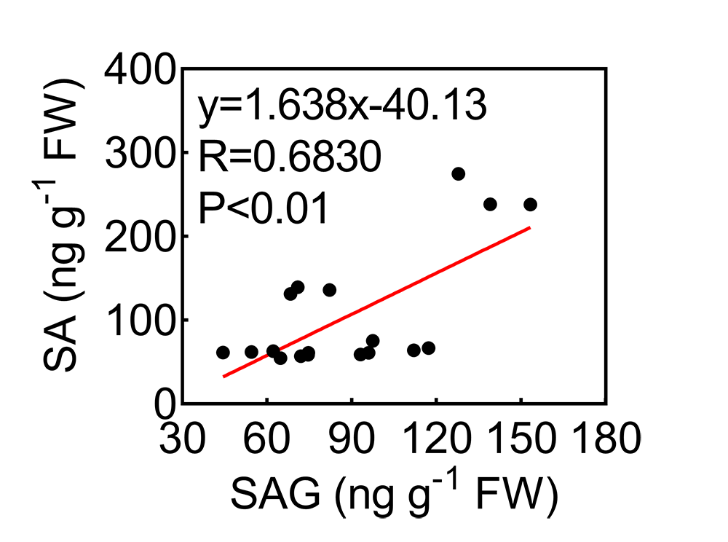
**

**Figure S3.** Correlation analysis between content of SAG and SA during peach fruit ripening.


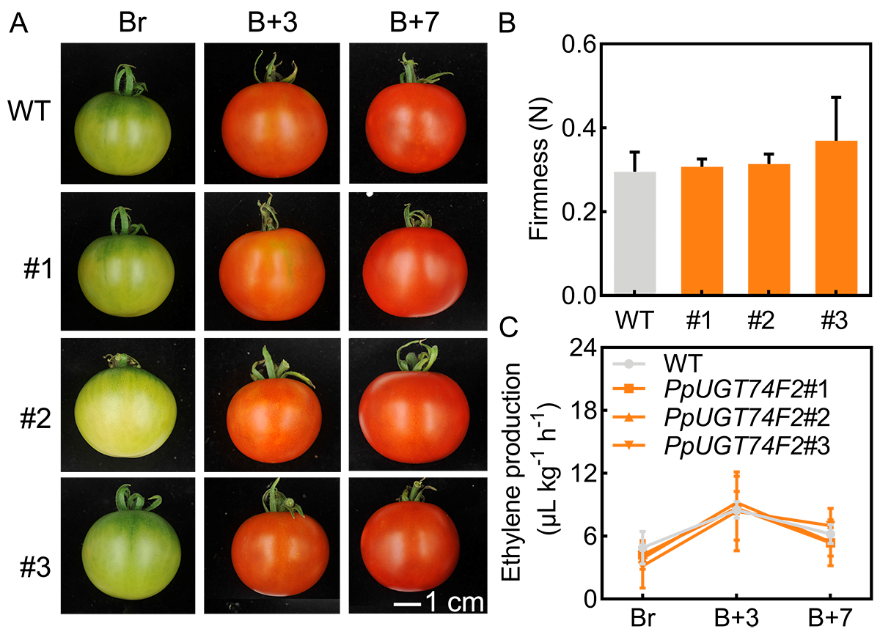


**Figure S4.** Phenotypes of tomatoes heterologously expressing *PpUGT74F2*. **A** Photograph of transgenic tomato fruit. The scale bar is applicable to each image. **B** Firmness of transgenic tomato fruit. Data are collected from 8 to 10 tomato fruit, and presented as mean value ± SD. **C** Ethylene production of transgenic tomato fruit. Data were collected from 9 to 12 tomato fruit, and presented as mean value ± SD.


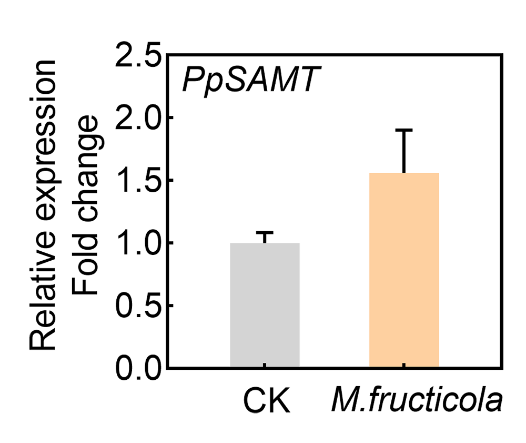


**Figure S5.** Expression of *PpSAMT* in peach fruit infected by *M. fructicola* after 3 dpi.

**
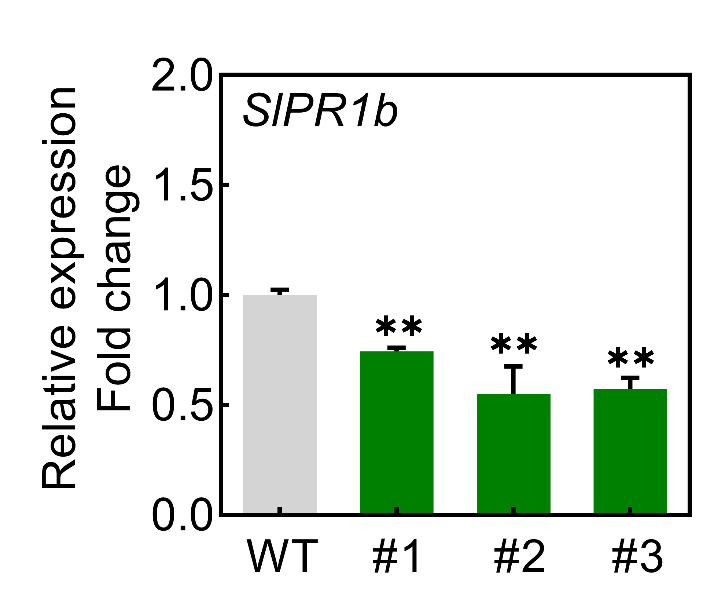
**

**Figure S6.** The expression of *SlPR1b* in transgenic tomato leaves with *Pst* DC3000 infection after 2 dpi.

**
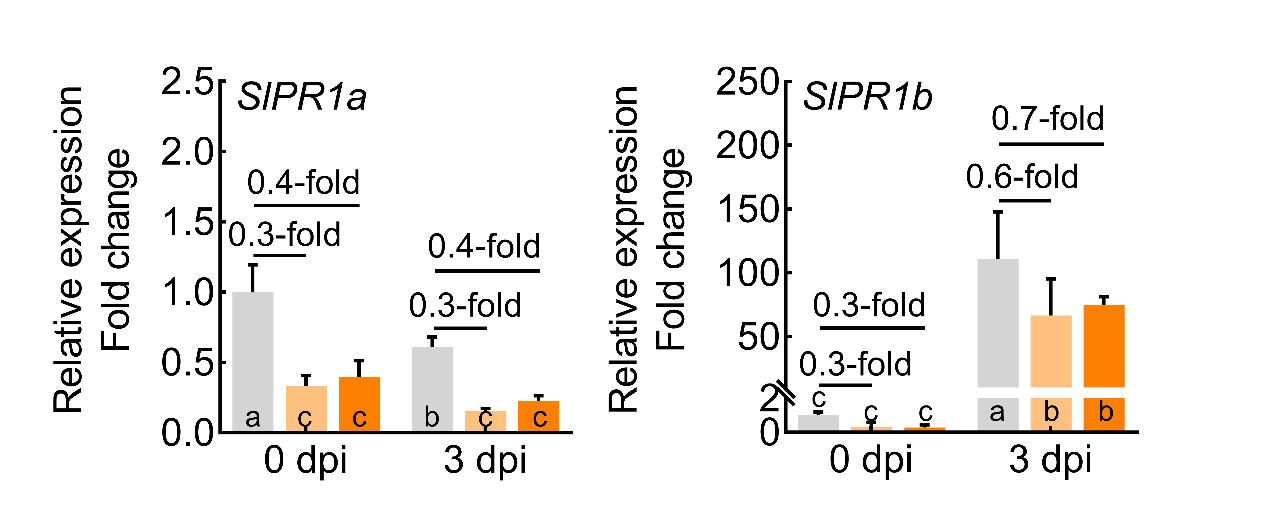
**

**Figure S7.** The expression of *SlPR1a* and *SlPR1b* in transgenic tomato fruit with *B. cinerea* infection at 0 dpi and 3 dpi.

**
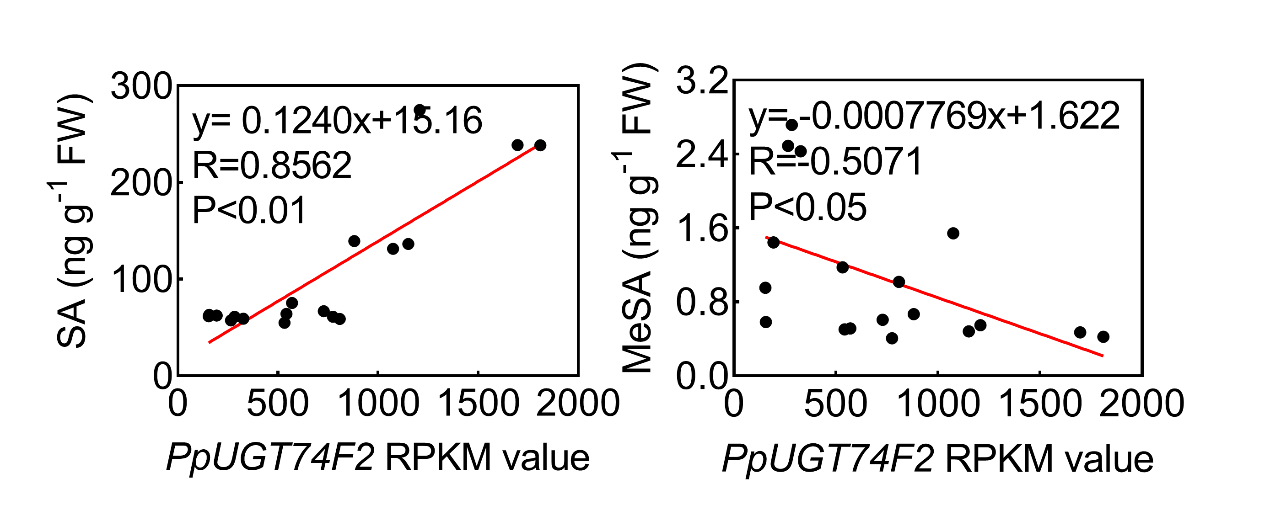
**

**Figure S8.** Correlation analysis between *PpUGT74F2* expression and SA, MeSA levels.

**Table S1**. RPKM of UGT genes during peach fruit ripening.

| GeneID | 34 DAB | 71 DAB | 94 DAB | 108 DAB | 111 DAB | 114 DAB |
| --- | --- | --- | --- | --- | --- | --- |
| **Prupe.7G267900** | 1571.46±320.05 | 1035.78±139.45 | 613.85±100.46 | 705.54±150.51 | 292.25±31.55 | 168.24±22.71 |
| Prupe.2G184800 | 139.81±50.39 | 28.64±9.44 | 17.83±1.04 | 3.21±1.53 | 0.57±0.32 | 0.37±0.11 |
| Prupe.5G116100 | 65.52±10.91 | 39.43±4.79 | 2.87±0.43 | 1.18±0.26 | 2.89±1.25 | 1.66±0.73 |
| Prupe.2G085000 | 79.73±25.13 | 607.20±124.95 | 464.36±76.71 | 888.67±115.63 | 383.92±133.50 | 323.50±16.94 |
| Prupe.3G184900 | 50.80±14.62 | 24.51±1.43 | 3.61±0.59 | 8.60±4.72 | 11.24±1.42 | 9.14±1.00 |
| Prupe.1G519600 | 87.50±28.33 | 137.87±58.13 | 318.09±18.60 | 647.16±23.95 | 1129.30±130.37 | 1231.88±71.21 |
| Prupe.2G085300 | 78.57±26.98 | 356.01±34.02 | 122.81±16.96 | 31.20±6.23 | 29.95±5.65 | 25.01±4.03 |
| Prupe.1G519500 | 119.22±78.01 | 31.12±4.07 | 31.12±4.68 | 89.89±20.52 | 42.49±6.15 | 26.21±4.15 |
| Prupe.5G178200 | 27.46±5.89 | 12.19±0.28 | 5.45±1.21 | 2.35±0.43 | 0.97±0.06 | 1.05±0.33 |
| Prupe.8G186000 | 33.59±6.63 | 52.35±1.05 | 61.25±10.10 | 10.89±4.53 | 2.20±0.46 | 1.39±0.33 |
| Prupe.3G184400 | 29.23±4.87 | 14.41±2.43 | 1.21±0.53 | 0.35±0.25 | 0.03±0.06 | 0.01±0.01 |
| Prupe.5G203300 | 13.78±0.57 | 16.89±0.51 | 17.63±0.73 | 17.27±1.11 | 14.52±0.69 | 13.64±1.83 |
| Prupe.3G184800 | 10.07±0.62 | 11.43±1.80 | 3.63±0.48 | 5.86±1.55 | 7.38±0.90 | 6.37±0.75 |
| Prupe.5G022600 | 10.67±4.62 | 42.67±42.32 | 13.06±5.85 | 38.46±11.23 | 76.95±24.81 | 109.40±25.84 |
| Prupe.7G149100 | 24.58±6.23 | 23.95±2.89 | 18.09±1.35 | 21.06±6.14 | 13.46±3.09 | 11.31±1.48 |
| Prupe.8G130300 | 11.45±3.74 | 9.18±2.14 | 6.50±4.76 | 11.31±4.08 | 22.19±12.67 | 23.34±9.15 |
| Prupe.1G505100 | 20.73±4.14 | 12.90±1.40 | 9.58±1.23 | 9.18±1.37 | 3.75±0.07 | 2.45±0.42 |
| Prupe.6G050000 | 19.37±2.46 | 11.06±2.35 | 5.16±1.60 | 4.64±3.18 | 0.37±0.16 | 0.28±0.04 |
| Prupe.3G282700 | 16.09±0.50 | 13.60±1.02 | 1.27±0.35 | 0.42±0.29 | 0.17±0.02 | 0.11±0.03 |
| Prupe.8G185900 | 8.43±2.71 | 1.37±0.25 | 0.66±0.20 | 0.27±0.04 | 0.06±0.07 | 0.03±0.02 |
| Prupe.1G554400 | 13.17±3.78 | 14.92±0.21 | 18.07±1.02 | 17.51±3.78 | 12.03±2.51 | 10.77±2.37 |
| Prupe.6G228400 | 11.79±1.01 | 2.27±0.22 | 0.99±0.16 | 0.77±0.09 | 2.19±1.23 | 2.22±1.04 |
| Prupe.4G117900 | 13.72±1.97 | 20.87±1.51 | 39.88±20.46 | 73.93±6.21 | 81.96±2.50 | 57.82±14.07 |
| Prupe.3G184600 | 3.29±3.28 | 3.00±0.51 | 2.52±1.32 | 5.47±2.50 | 7.36±5.43 | 9.47±4.10 |

**Table S1** Continued

| GeneID | 34 DAB | 71 DAB | 94 DAB | 108 DAB | 111 DAB | 114 DAB |
| --- | --- | --- | --- | --- | --- | --- |
| Prupe.6G189800 | 10.34±0.86 | 9.30±0.55 | 2.59±0.61 | 1.58±0.20 | 0.46±0.30 | 0.17±0.02 |
| Prupe.2G303000 | 6.91±0.98 | 7.86±1.07 | 2.83±0.25 | 5.80±0.35 | 14.03±6.71 | 17.72±7.71 |
| Prupe.6G058900 | 6.41±0.06 | 7.39±1.89 | 1.65±0.20 | 0.84±0.20 | 0.49±0.20 | 0.31±0.02 |
| Prupe.8G129800 | 10.96±1.47 | 6.11±1.10 | 0.59±0.24 | 0.22±0.13 | 0.05±0.08 | 0.00±0.00 |
| Prupe.I001100 | 8.98±0.77 | 48.71±7.55 | 7.12±2.27 | 3.02±2.05 | 0.32±0.35 | 0.06±0.03 |
| Prupe.3G256000 | 8.73±0.33 | 3.10±0.79 | 0.85±0.09 | 0.19±0.08 | 0.05±0.08 | 0.00±0.00 |
| Prupe.6G008300 | 7.00±0.88 | 4.90±1.01 | 1.16±0.22 | 1.26±0.14 | 0.83±0.26 | 0.50±0.11 |
| Prupe.8G063500 | 6.74±0.38 | 4.76±0.37 | 1.29±0.15 | 0.25±0.04 | 0.40±0.15 | 0.17±0.07 |
| Prupe.6G233400 | 8.32±2.11 | 25.82±3.85 | 30.64±13.02 | 0.86±0.43 | 0.13±0.12 | 0.06±0.03 |
| Prupe.I002600 | 19.86±5.28 | 39.35±3.41 | 15.03±4.72 | 3.88±2.23 | 0.58±0.68 | 0.61±0.78 |
| Prupe.I001000 | 5.04±1.00 | 4.29±1.16 | 5.54±1.63 | 9.84±1.76 | 7.38±2.34 | 6.86±1.11 |
| Prupe.6G008000 | 8.15±1.87 | 1.75±0.31 | 0.58±0.18 | 0.21±0.09 | 0.19±0.08 | 0.09±0.05 |
| Prupe.8G150600 | 3.89±1.46 | 3.30±0.89 | 3.78±0.92 | 7.43±1.80 | 5.46±0.60 | 5.26±0.65 |
| Prupe.3G260200 | 4.99±1.81 | 1.86±0.91 | 3.60±2.30 | 7.36±0.89 | 3.27±1.41 | 2.27±1.42 |
| Prupe.6G059000 | 4.59±1.30 | 11.77±3.79 | 9.19±1.89 | 15.46±1.49 | 16.29±2.42 | 17.94±4.33 |
| Prupe.5G210800 | 3.09±0.42 | 4.02±0.86 | 9.17±2.11 | 10.29±1.99 | 8.85±0.66 | 10.82±1.31 |
| Prupe.1G520000 | 2.09±0.34 | 1.97±0.24 | 0.39±0.11 | 0.19±0.05 | 0.20±0.05 | 0.10±0.01 |
| Prupe.7G124100 | 3.72±1.68 | 5.57±0.90 | 1.33±0.26 | 1.29±0.36 | 1.36±0.10 | 1.16±0.04 |
| Prupe.3G189600 | 2.50±0.61 | 0.31±0.16 | 0.14±0.13 | 0.01±0.01 | 0.00±0.00 | 0.01±0.01 |
| Prupe.1G552100 | 4.77±0.58 | 5.49±0.61 | 5.10±0.44 | 4.62±0.85 | 1.73±0.47 | 1.24±0.18 |
| Prupe.1G295300 | 3.16±0.98 | 5.27±0.65 | 2.58±0.83 | 2.54±0.24 | 1.29±0.26 | 1.57±0.54 |
| Prupe.1G169100 | 1.99±1.65 | 0.57±0.22 | 0.63±0.18 | 1.55±0.58 | 2.83±0.38 | 2.47±0.79 |
| Prupe.1G505200 | 3.19±0.69 | 2.65±0.54 | 8.92±2.10 | 8.84±1.19 | 4.44±0.67 | 3.03±1.37 |
| Prupe.1G520400 | 2.41±0.42 | 1.13±0.54 | 0.24±0.16 | 0.53±0.20 | 0.21±0.02 | 0.31±0.11 |

**Table S1** Continued

| GeneID | 34 DAB | 71 DAB | 94 DAB | 108 DAB | 111 DAB | 114 DAB |
| --- | --- | --- | --- | --- | --- | --- |
| Prupe.6G136900 | 2.47±0.74 | 4.20±0.81 | 2.69±0.45 | 2.34±0.40 | 1.19±0.53 | 0.87±0.25 |
| Prupe.3G185000 | 1.75±1.09 | 1.52±0.38 | 0.62±0.13 | 0.34±0.36 | 0.83±0.24 | 0.93±0.15 |
| Prupe.8G186200 | 1.26±0.92 | 0.14±0.04 | 0.01±0.02 | 0.08±0.02 | 0.03±0.04 | 0.02±0.02 |
| Prupe.6G189900 | 3.20±1.48 | 0.25±0.03 | 0.21±0.11 | 0.26±0.29 | 0.15±0.24 | 0.00±0.00 |
| Prupe.1G547500 | 2.33±0.33 | 2.35±0.14 | 15.60±7.99 | 33.12±5.23 | 33.73±3.44 | 29.26±0.48 |
| Prupe.1G519800 | 1.07±0.20 | 1.93±0.16 | 1.39±0.01 | 1.27±0.09 | 1.05±0.05 | 1.08±0.17 |
| Prupe.6G233100 | 1.62±0.21 | 1.27±0.25 | 1.71±1.00 | 1.77±0.59 | 0.80±0.13 | 0.40±0.15 |
| Prupe.2G243600 | 0.72±0.97 | 1.07±1.05 | 0.68±0.77 | 2.81±0.97 | 3.53±1.41 | 3.67±2.38 |
| Prupe.1G169300 | 1.43±0.33 | 0.33±0.14 | 1.56±0.65 | 6.11±3.88 | 8.64±1.96 | 10.36±4.36 |
| Prupe.6G050200 | 1.30±0.41 | 3.29±0.54 | 1.90±0.44 | 0.44±0.33 | 0.06±0.04 | 0.09±0.12 |
| Prupe.7G124400 | 1.40±0.38 | 0.62±0.25 | 0.16±0.10 | 0.55±0.21 | 0.79±0.08 | 0.46±0.02 |
| Prupe.8G150700 | 1.17±0.21 | 0.10±0.03 | 0.18±0.19 | 0.11±0.06 | 0.01±0.02 | 0.00±0.00 |
| Prupe.8G129700 | 2.00±0.38 | 1.08±0.35 | 0.35±0.19 | 0.03±0.03 | 0.01±0.02 | 0.02±0.02 |
| Prupe.6G008400 | 1.46±0.34 | 0.69±0.35 | 0.10±0.07 | 0.03±0.01 | 0.02±0.02 | 0.00±0.01 |
| Prupe.6G233600 | 1.62±0.60 | 10.39±1.52 | 2.95±0.94 | 1.14±0.49 | 0.11±0.05 | 0.03±0.03 |
| Prupe.1G053300 | 0.94±0.57 | 0.06±0.06 | 0.02±0.04 | 0.03±0.06 | 0.00±0.00 | 0.00±0.00 |
| Prupe.6G049400 | 4.16±1.68 | 6.51±1.43 | 9.55±2.66 | 9.66±2.51 | 4.91±0.76 | 4.25±0.47 |
| Prupe.2G184700 | 1.05±0.21 | 0.11±0.03 | 0.08±0.06 | 0.02±0.04 | 0.00±0.00 | 0.00±0.00 |
| Prupe.I000900 | 0.67±0.28 | 0.46±0.22 | 0.52±0.29 | 0.28±0.10 | 0.04±0.08 | 0.05±0.05 |
| Prupe.2G243500 | 0.33±0.33 | 0.12±0.16 | 0.06±0.06 | 0.39±0.17 | 0.33±0.06 | 0.30±0.26 |
| Prupe.3G190400 | 0.71±0.17 | 5.82±1.47 | 8.18±1.21 | 4.76±0.50 | 2.11±0.72 | 1.79±0.28 |
| Prupe.7G149200 | 0.27±0.25 | 0.04±0.05 | 0.13±0.07 | 0.75±0.18 | 1.11±0.22 | 1.39±0.48 |
| Prupe.6G233200 | 1.07±0.19 | 0.93±0.23 | 5.66±1.67 | 10.13±2.10 | 8.18±1.03 | 4.44±1.02 |
| Prupe.8G186100 | 0.37±0.30 | 0.61±0.05 | 0.40±0.22 | 0.13±0.13 | 0.10±0.06 | 0.11±0.01 |

**Table S1** Continued

| GeneID | 34 DAB | 71 DAB | 94 DAB | 108 DAB | 111 DAB | 114 DAB |
| --- | --- | --- | --- | --- | --- | --- |
| Prupe.2G101800 | 0.62±0.31 | 0.82±0.17 | 0.24±0.10 | 0.24±0.14 | 0.07±0.05 | 0.04±0.04 |
| Prupe.2G085800 | 1.80±0.39 | 0.03±0.00 | 0.00±0.00 | 0.00±0.00 | 0.00±0.00 | 0.00±0.00 |
| Prupe.7G124300 | 0.80±0.22 | 0.54±0.14 | 0.13±0.03 | 0.30±0.13 | 0.42±0.20 | 0.30±0.07 |
| Prupe.5G116200 | 0.24±0.23 | 0.09±0.09 | 0.02±0.02 | 0.05±0.06 | 0.15±0.02 | 0.10±0.05 |
| Prupe.3G189300 | 0.43±0.14 | 0.10±0.06 | 0.05±0.03 | 0.14±0.14 | 0.21±0.06 | 0.15±0.15 |
| Prupe.1G179700 | 0.34±0.05 | 0.46±0.12 | 0.71±0.09 | 0.05±0.02 | 0.00±0.01 | 0.00±0.00 |
| Prupe.6G233300 | 0.52±0.06 | 1.27±0.16 | 2.14±1.05 | 0.15±0.10 | 0.14±0.13 | 0.04±0.04 |
| Prupe.7G124600 | 0.51±0.10 | 0.79±0.09 | 0.49±0.14 | 0.23±0.22 | 0.45±0.19 | 0.52±0.13 |
| Prupe.1G554300 | 0.50±0.08 | 0.56±0.16 | 0.74±0.36 | 0.50±0.07 | 0.37±0.13 | 0.35±0.11 |
| Prupe.7G124200 | 0.20±0.16 | 0.32±0.07 | 0.23±0.12 | 1.48±0.36 | 4.85±0.71 | 4.60±1.62 |
| Prupe.1G519700 | 0.50±0.18 | 1.43±1.57 | 0.78±0.37 | 0.43±0.30 | 0.10±0.04 | 0.08±0.04 |
| Prupe.1G266300 | 0.37±0.14 | 1.27±0.19 | 1.75±0.45 | 1.28±0.83 | 0.72±0.52 | 0.14±0.02 |
| Prupe.1G053400 | 0.39±0.10 | 0.22±0.11 | 0.04±0.05 | 0.01±0.02 | 0.00±0.00 | 0.00±0.00 |
| Prupe.2G085100 | 0.18±0.18 | 0.99±1.03 | 3.55±1.63 | 72.93±13.30 | 78.60±28.09 | 84.47±1.53 |
| Prupe.3G260100 | 0.18±0.06 | 0.04±0.04 | 0.02±0.04 | 0.06±0.02 | 0.01±0.02 | 0.01±0.02 |
| Prupe.1G282300 | 0.29±0.08 | 0.00±0.00 | 0.00±0.00 | 0.00±0.00 | 0.00±0.00 | 0.00±0.00 |
| Prupe.1G534200 | 0.19±0.10 | 0.16±0.09 | 0.09±0.06 | 0.29±0.15 | 0.05±0.02 | 0.07±0.08 |
| Prupe.3G190600 | 0.15±0.03 | 0.18±0.07 | 0.37±0.12 | 0.42±0.18 | 0.20±0.04 | 0.18±0.05 |
| Prupe.6G050100 | 0.20±0.06 | 0.27±0.15 | 0.30±0.05 | 0.09±0.08 | 0.00±0.00 | 0.00±0.00 |
| Prupe.1G520300 | 0.15±0.10 | 0.08±0.08 | 0.00±0.00 | 0.00±0.00 | 0.00±0.00 | 0.00±0.00 |
| Prupe.6G266600 | 0.05±0.07 | 0.07±0.07 | 0.00±0.00 | 0.64±0.17 | 0.13±0.16 | 0.08±0.07 |
| Prupe.1G519900 | 0.15±0.04 | 0.62±0.23 | 0.60±0.16 | 1.12±0.20 | 0.71±0.14 | 0.56±0.25 |
| Prupe.2G084900 | 0.15±0.07 | 0.82±0.39 | 0.70±0.10 | 1.01±0.05 | 0.70±0.26 | 0.56±0.07 |
| Prupe.3G189700 | 0.04±0.04 | 0.01±0.01 | 0.00±0.00 | 0.00±0.00 | 0.01±0.01 | 0.02±0.01 |

**Table S1** Continued

| GeneID | 34 DAB | 71 DAB | 94 DAB | 108 DAB | 111 DAB | 114 DAB |
| --- | --- | --- | --- | --- | --- | --- |
| Prupe.1G520200 | 0.10±0.04 | 0.07±0.01 | 0.00±0.00 | 0.00±0.00 | 0.00±0.00 | 0.00±0.00 |
| Prupe.1G458200 | 0.04±0.06 | 0.00±0.00 | 0.00±0.00 | 0.00±0.00 | 0.00±0.00 | 0.00±0.00 |
| Prupe.2G085200 | 0.19±0.07 | 0.01±0.01 | 0.00±0.00 | 0.00±0.00 | 0.01±0.01 | 0.03±0.05 |
| Prupe.6G049600 | 0.10±0.08 | 0.19±0.06 | 0.07±0.03 | 0.08±0.02 | 0.09±0.10 | 0.02±0.03 |
| Prupe.6G190100 | 0.14±0.07 | 0.04±0.04 | 0.00±0.00 | 0.02±0.04 | 0.00±0.00 | 0.02±0.02 |
| Prupe.3G256200 | 0.03±0.03 | 0.23±0.12 | 0.56±0.24 | 0.89±0.13 | 0.38±0.15 | 0.35±0.12 |
| Prupe.1G091100 | 0.08±0.05 | 0.28±0.03 | 0.23±0.03 | 0.06±0.03 | 0.02±0.01 | 0.00±0.00 |
| Prupe.3G189500 | 0.33±0.48 | 0.12±0.06 | 0.00±0.00 | 0.01±0.02 | 0.00±0.00 | 0.00±0.00 |
| Prupe.7G124500 | 0.06±0.05 | 0.06±0.05 | 0.00±0.00 | 0.01±0.02 | 0.08±0.04 | 0.06±0.08 |
| Prupe.7G055200 | 0.03±0.05 | 0.27±0.08 | 1.27±0.35 | 0.85±0.29 | 0.36±0.16 | 0.33±0.12 |
| Prupe.6G190300 | 0.63±0.51 | 0.62±0.26 | 0.01±0.02 | 0.10±0.12 | 0.47±0.08 | 0.50±0.02 |
| Prupe.2G324700 | 0.07±0.03 | 0.14±0.08 | 4.42±4.45 | 24.80±28.73 | 31.46±19.61 | 32.67±7.34 |
| Prupe.2G086000 | 0.03±0.03 | 0.00±0.00 | 0.00±0.00 | 0.00±0.00 | 0.00±0.00 | 0.00±0.00 |
| Prupe.8G130800 | 0.02±0.03 | 0.02±0.04 | 0.07±0.11 | 0.06±0.08 | 0.07±0.09 | 0.04±0.04 |
| Prupe.6G203500 | 0.08±0.04 | 0.03±0.03 | 0.01±0.02 | 0.00±0.00 | 0.00±0.00 | 0.00±0.00 |
| Prupe.3G190500 | 0.03±0.01 | 0.16±0.12 | 0.18±0.05 | 0.09±0.04 | 0.01±0.02 | 0.03±0.02 |
| Prupe.8G130700 | 0.02±0.02 | 0.00±0.00 | 0.00±0.00 | 0.00±0.00 | 0.02±0.03 | 0.00±0.00 |
| Prupe.6G008600 | 0.02±0.02 | 0.02±0.02 | 0.05±0.02 | 0.03±0.03 | 0.01±0.01 | 0.01±0.01 |
| Prupe.7G013100 | 0.07±0.05 | 0.13±0.04 | 0.13±0.14 | 0.05±0.04 | 0.04±0.00 | 0.05±0.05 |
| Prupe.6G007800 | 0.13±0.12 | 0.01±0.02 | 0.05±0.04 | 0.02±0.04 | 0.02±0.02 | 0.00±0.00 |
| Prupe.8G130400 | 0.02±0.03 | 0.00±0.00 | 0.00±0.00 | 0.00±0.00 | 0.00±0.00 | 0.00±0.00 |
| Prupe.1G181000 | 0.08±0.04 | 0.05±0.02 | 0.03±0.03 | 0.17±0.24 | 0.00±0.00 | 0.01±0.02 |
| Prupe.7G124000 | 0.02±0.02 | 0.49±0.70 | 0.13±0.15 | 0.12±0.08 | 0.08±0.05 | 0.06±0.02 |
| Prupe.8G135800 | 0.04±0.00 | 0.12±0.13 | 0.00±0.00 | 0.00±0.00 | 0.01±0.02 | 0.00±0.00 |

**Table S1** Continued

| GeneID | 34 DAB | 71 DAB | 94 DAB | 108 DAB | 111 DAB | 114 DAB |
| --- | --- | --- | --- | --- | --- | --- |
| Prupe.2G175000 | 0.03±0.03 | 0.01±0.02 | 0.00±0.00 | 0.00±0.00 | 0.00±0.00 | 0.00±0.00 |
| Prupe.1G547700 | 0.04±0.04 | 0.06±0.08 | 0.03±0.03 | 0.02±0.03 | 0.06±0.05 | 0.04±0.01 |
| Prupe.4G054800 | 0.11±0.11 | 0.02±0.03 | 0.07±0.06 | 0.01±0.02 | 0.00±0.00 | 0.00±0.00 |
| Prupe.3G190800 | 0.03±0.03 | 0.07±0.03 | 0.07±0.03 | 0.04±0.04 | 0.04±0.01 | 0.02±0.02 |
| Prupe.3G185400 | 0.04±0.02 | 0.09±0.04 | 0.11±0.12 | 0.04±0.07 | 0.00±0.00 | 0.00±0.00 |
| Prupe.6G203800 | 0.05±0.05 | 0.20±0.05 | 0.48±0.20 | 0.34±0.11 | 0.45±0.06 | 0.19±0.14 |
| Prupe.3G190100 | 0.01±0.01 | 0.26±0.07 | 1.25±0.40 | 6.22±3.08 | 5.11±2.90 | 4.71±0.21 |
| Prupe.8G130100 | 0.01±0.01 | 0.00±0.00 | 0.00±0.00 | 0.00±0.00 | 0.00±0.00 | 0.01±0.02 |
| Prupe.1G382700 | 0.02±0.02 | 0.00±0.00 | 0.00±0.00 | 0.00±0.00 | 0.00±0.00 | 0.00±0.00 |
| Prupe.6G203900 | 0.06±0.09 | 0.07±0.07 | 0.45±0.22 | 0.44±0.10 | 0.43±0.20 | 0.23±0.11 |
| Prupe.1G179400 | 0.00±0.00 | 0.00±0.00 | 0.00±0.00 | 0.00±0.00 | 0.01±0.02 | 0.00±0.00 |
| Prupe.2G243700 | 0.00±0.00 | 0.03±0.04 | 0.09±0.06 | 0.12±0.04 | 0.03±0.04 | 0.08±0.08 |
| Prupe.3G188500 | 0.00±0.00 | 0.01±0.02 | 0.02±0.02 | 0.11±0.06 | 0.06±0.05 | 0.02±0.02 |
| Prupe.2G174700 | 0.00±0.00 | 0.02±0.02 | 0.02±0.04 | 0.25±0.15 | 0.00±0.00 | 0.00±0.00 |
| Prupe.8G130900 | 0.00±0.00 | 0.19±0.11 | 0.18±0.15 | 0.01±0.01 | 0.02±0.02 | 0.03±0.05 |
| Prupe.1G547800 | 0.05±0.05 | 0.08±0.04 | 0.02±0.02 | 0.17±0.15 | 0.40±0.21 | 0.12±0.06 |
| Prupe.6G008700 | 0.02±0.03 | 0.01±0.01 | 0.02±0.01 | 0.00±0.00 | 0.00±0.00 | 0.00±0.00 |
| Prupe.1G053200 | 0.06±0.07 | 0.06±0.04 | 0.13±0.07 | 0.09±0.06 | 0.04±0.02 | 0.02±0.02 |
| Prupe.8G130200 | 0.02±0.02 | 0.08±0.04 | 0.10±0.10 | 0.38±0.25 | 2.03±0.59 | 2.23±0.43 |
| Prupe.1G520100 | 0.00±0.00 | 0.00±0.00 | 0.00±0.00 | 0.03±0.05 | 0.02±0.02 | 0.00±0.00 |
| Prupe.6G049700 | 0.01±0.02 | 0.03±0.02 | 0.00±0.00 | 0.01±0.01 | 0.00±0.00 | 0.00±0.00 |
| Prupe.8G130500 | 0.00±0.00 | 0.00±0.00 | 0.00±0.00 | 0.01±0.01 | 0.00±0.00 | 0.00±0.00 |
| Prupe.6G015900 | 0.00±0.00 | 0.01±0.02 | 0.04±0.04 | 0.00±0.00 | 0.01±0.02 | 0.01±0.02 |
| Prupe.6G049300 | 0.00±0.00 | 0.05±0.05 | 0.70±0.36 | 1.60±0.13 | 0.82±0.29 | 0.58±0.45 |

**Table S1** Continued

| GeneID | 34 DAB | 71 DAB | 94 DAB | 108 DAB | 111 DAB | 114 DAB |
| --- | --- | --- | --- | --- | --- | --- |
| Prupe.3G185100 | 0.00±0.00 | 0.00±0.00 | 0.00±0.00 | 0.00±0.00 | 0.00±0.00 | 0.01±0.02 |
| Prupe.2G174800 | 0.00±0.00 | 0.00±0.00 | 0.00±0.00 | 0.01±0.02 | 0.00±0.00 | 0.00±0.00 |
| Prupe.1G090500 | 0.02±0.04 | 0.00±0.00 | 0.00±0.00 | 0.00±0.00 | 0.12±0.20 | 0.01±0.02 |
| Prupe.1G458400 | 0.00±0.00 | 0.01±0.02 | 0.00±0.00 | 0.00±0.00 | 0.00±0.00 | 0.00±0.00 |
| Prupe.8G130600 | 0.00±0.00 | 0.00±0.00 | 0.00±0.00 | 0.00±0.00 | 0.03±0.03 | 0.01±0.02 |
| Prupe.3G166800 | 0.01±0.03 | 0.01±0.02 | 0.01±0.02 | 0.03±0.02 | 0.07±0.02 | 0.15±0.07 |
| Prupe.3G187400 | 0.00±0.00 | 0.01±0.01 | 0.01±0.02 | 0.00±0.00 | 0.00±0.00 | 0.00±0.00 |
| Prupe.6G265900 | 0.00±0.00 | 0.09±0.13 | 0.00±0.00 | 0.07±0.09 | 0.01±0.02 | 0.02±0.03 |
| Prupe.3G256100 | 0.00±0.00 | 0.00±0.00 | 0.03±0.05 | 0.05±0.05 | 0.00±0.00 | 0.01±0.02 |
| Prupe.4G173600 | 0.00±0.00 | 0.00±0.00 | 0.00±0.00 | 0.01±0.02 | 0.00±0.00 | 0.00±0.00 |
| Prupe.1G053500 | 0.00±0.00 | 0.01±0.01 | 0.00±0.00 | 0.00±0.00 | 0.00±0.00 | 0.00±0.00 |
| Prupe.3G189100 | 0.00±0.00 | 0.00±0.00 | 0.00±0.00 | 0.00±0.00 | 0.01±0.02 | 0.00±0.00 |
| Prupe.1G169200 | 0.00±0.00 | 0.01±0.02 | 0.00±0.00 | 0.15±0.09 | 0.68±0.43 | 0.76±0.30 |
| Prupe.6G233500 | 0.01±0.02 | 0.02±0.03 | 0.04±0.01 | 0.00±0.00 | 0.00±0.00 | 0.01±0.02 |
| Prupe.3G256300 | 0.00±0.00 | 0.00±0.00 | 0.04±0.05 | 0.29±0.01 | 0.06±0.04 | 0.07±0.02 |
| Prupe.6G108000 | 0.00±0.00 | 0.01±0.02 | 0.00±0.00 | 0.02±0.04 | 0.00±0.00 | 0.00±0.00 |
| Prupe.3G188700 | 0.00±0.00 | 0.00±0.00 | 0.00±0.00 | 0.00±0.00 | 0.03±0.05 | 0.00±0.00 |
| Prupe.3G188600 | 0.00±0.00 | 0.00±0.00 | 0.05±0.02 | 0.04±0.04 | 0.01±0.02 | 0.00±0.00 |
| Prupe.8G131000 | 0.75±0.26 | 1.50±0.03 | 0.98±0.37 | 0.40±0.13 | 0.04±0.04 | 0.00±0.00 |
| Prupe.6G007700 | 0.02±0.01 | 0.01±0.01 | 0.00±0.00 | 0.00±0.00 | 0.00±0.00 | 0.00±0.00 |
| Prupe.6G267000 | 0.01±0.01 | 0.00±0.00 | 0.00±0.00 | 0.00±0.00 | 0.00±0.00 | 0.00±0.00 |
| Prupe.3G190300 | 0.00±0.00 | 0.00±0.00 | 0.01±0.02 | 0.00±0.00 | 0.00±0.00 | 0.00±0.00 |
| Prupe.1G180900 | 0.00±0.00 | 0.00±0.00 | 0.01±0.01 | 0.00±0.00 | 0.00±0.00 | 0.00±0.00 |

**Table S2**. Identity (%) of amino acid sequences between PpUGT74F2, AtUGT74F1, AtUGT74F2, AtUGT76B1, AtUGT71C3 and CsUGT87E7.

|  | PpUGT74F2 | AtUGT74F1 | AtUGT74F2 | AtUGT76B1 | AtUGT71C3 | CsUGT87E7 |
| --- | --- | --- | --- | --- | --- | --- |
| PpUGT74F2 | 100 |  |  |  |  |  |
| AtUGT74F1 | 51.5 | 100 |  |  |  |  |
| AtUGT74F2 | 53.6 | 76.6 | 100 |  |  |  |
| AtUGT76B1 | 22.11 | 25.43 | 24.79 | 100 |  |  |
| AtUGT71C3 | 23.05 | 21.99 | 24.39 | 22.45 | 100 |  |
| CsUGT87E7 | 7.26 | 8.61 | 8.02 | 6.71 | 8.39 | 100 |

**Table S3**. The enzyme activity analysis of PpUGT74F2 towards chemical substrates.

|  | Substrate | Activity of PpUGT74F2 |
| --- | --- | --- |
| Hormones | Salicylic acid | **+** |
|  | Indoleacetic acid | **-** |
|  | Jasmonate | **-** |
|  | Abscisic acid | **-** |
|  | Brassinosteroid | **-** |
|  | Zeatin | **-** |
|  | 3-Indole butyric acid | **-** |
| Flavonoids | Quercetin | **-** |
|  | Myricetin | **-** |
|  | Kaempferol | **-** |
|  | Isorhamnetin | **-** |
|  | Catechins | **-** |
|  | Epicatechin | **-** |
|  | Naringin | **-** |
|  | Hesperidin | **-** |
|  | Luteolin | **-** |
|  | Celery | **-** |
|  | Baicalein | **-** |
|  | Genistein | **-** |
|  | Daidzein | **-** |
| Volatiles | Methyl Salicylate | **+** |
|  | α-Terpineol | **-** |
|  | Linalool | **-** |
|  | Geraniol | **-** |
|  | Eugenol | **-** |
|  | 2-Phenylethanol | **-** |
|  | Benzyl alcohol | **-** |
| Anthocyanins | Mallow pigment | **-** |
|  | Cornflower | **-** |

**Table S4**. Primer sequences for constructing vector used in the present study.

| Primers | Sequence（5‘ -3’） | Description | Vector (restriction sites) |
| --- | --- | --- | --- |
| *PpUGT74F2*-SK-F | agaactagtggatccATGGATAGAGGCCACGTCTTGG | For overexpressing *PpGH28BG1* in peach fruit | pGreen II 002962-SK (BamHI and Sal I) |
| *PpUGT74F2*-SK-R | cccctcgaggtcgacAGCAGCTGCGGTTGTCAATTTG |  |  |
| pBI121-*PpUGT74F2*-F | acgggggactctagaATGGATAGAGGCCACGTCTT | For overexpressing *PpGH28BG1* in tomato | PBI121 (XbaI and BamHI) |
| pBI121-*PpUGT74F2*-R | accacccggggatccAGCAGCTGCGGTTGTCAATTTG |  |  |
| pGEX-GST-*PpUGT74F2*-F | gttccgcgtggatccATGGATAGAGGCCACGTCTT | For purifying protein | pGEX-4T-1 (BamHI and EcoRI) |
| pGEX-GST-*PpUGT74F2*-R | ccgctcgaggaattcTTAAGCAGCTGCGGTTGTCA |  |  |

**Table S5**. Primer sequences for RT-qPCR analysis used in the present study.

| Primers | Gene ID | Sequence（5‘ -3’） |
| --- | --- | --- |
| *PpTEF2*-F | Prupe.4G138700 | GGTGTGACGATGAAGAGTGATG |
| *PpTEF2*-R |  | TGAAGGAGAGGGAAGGTGAAAG |
| *SlACTIN*-F | Solyc11g005330 | TGTCCCTATTTACGAGGGTTATG |
| *SlACTIN*-R |  | CAGTTAAATCACGACCAGCAAGA |
| *PpUGT74F2*-F | Prupe.7G267900 | TCAAGGCCACATAAACCCCT |
| *PpUGT74F2*-R |  | CCTCATCGTAGCCATCGGAT |
| *PpPR2*-F | Prupe.7G051800 | GGCGGAGGGTCTTTGGAAAT |
| *PpPR2*-R |  | TGGAGTCCCTCCCTTCACAT |
| *PpPR4*-F | Prupe.6G141100 | GAGAGCCACGTACAACCTGT |
| *PpPR4*-R |  | GCAGAAGGCAGTCCATCCAT |
| *PpPR5*-F | Prupe.1G383700 | ACTTCGACGACTCGGGAAAC |
| *PpPR5*-R |  | GGTCGAACCTGTCGTGAACT |
| *PpPAL*-F | Prupe.2G211800 | AGGAGAACCAAGCAAGGTGG |
| *PpPAL*-R |  | GCTGTGTGAGGCAATGTGTG |
| *PpSAMT*-F | Prupe.8G093600 | TGCACCTACAACCAAGGAGTT |
| *PpSAMT*-R |  | AGGCCACTGTTAGCTTGCTT |
| *SlPR1a*-F | Solyc01g008620 | GGCAGGAACACCAAAGAAACCA |
| *SlPR1a*-R |  | TGGCCTCTGGTCAGGTTTAAAG |
| *SlPR1b*-F | Solyc00g174340 | CCGTGCAATTGTGGGTGTC |
| *SlPR1b*-R |  | GAGTTGCGCCAGACTACTTG |
| *SlSAMT*-F | Solyc09g091550 | GCATGAGAGCTGTGGCTGA |
| *SlSAMT*-R |  | GCCATACACTTGGAGACAATCT |
